# Minocycline Loaded Hybrid Composites Nanoparticles for Mesenchymal Stem Cells Differentiation into Osteogenesis

**DOI:** 10.3390/ijms17081222

**Published:** 2016-07-28

**Authors:** Allister Yingwei Tham, Chinnasamy Gandhimathi, Jayaraman Praveena, Jayarama Reddy Venugopal, Seeram Ramakrishna, Srinivasan Dinesh Kumar

**Affiliations:** 1 Cellular and Molecular Epigenetics Lab, Lee Kong Chian School of Medicine, Nanyang Technological University, Singapore 636921, Singapore; THAM0115@e.ntu.edu.sg (A.Y.T.); CGandhimathi@ntu.edu.sg (C.G.); jayaraman.p@ntu.edu.sg (J.P.); 2 Center for Nanofibers and Nanotechnology, Department of Mechanical Engineering, National University of Singapore, Singapore 119260, Singapore; mpejrv@nus.edu.sg (J.R.V.); seeram@nus.edu.sg (S.R.)

**Keywords:** electrospraying, polycaprolactone, silk fibroin, hyaluronic acid, minocycline hydrochloride, nanoparticles

## Abstract

Bone transplants are used to treat fractures and increase new tissue development in bone tissue engineering. Grafting of massive implantations showing slow curing rate and results in cell death for poor vascularization. The potentials of biocomposite scaffolds to mimic extracellular matrix (ECM) and including new biomaterials could produce a better substitute for new bone tissue formation. A purpose of this study is to analyze polycaprolactone/silk fibroin/hyaluronic acid/minocycline hydrochloride (PCL/SF/HA/MH) nanoparticles initiate human mesenchymal stem cells (MSCs) proliferation and differentiation into osteogenesis. Electrospraying technique was used to develop PCL, PCL/SF, PCL/SF/HA and PCL/SF/HA/MH hybrid biocomposite nanoparticles and characterization was analyzed by field emission scanning electron microscope (FESEM), contact angle and Fourier transform infrared spectroscopy (FT-IR). The obtained results proved that the particle diameter and water contact angle obtained around 0.54 ± 0.12 to 3.2 ± 0.18 µm and 43.93 ± 10.8° to 133.1 ± 12.4° respectively. The cell proliferation and cell-nanoparticle interactions analyzed using (3-(4,5-dimethyl thiazol-2-yl)-5-(3-carboxymethoxyphenyl)-2-(4-sulfophenyl)-2*H*-tetrazolium inner salt) MTS assay (Promega, Madison, WI, USA), FESEM for cell morphology and 5-Chloromethylfluorescein diacetate (CMFDA) dye for imaging live cells. Osteogenic differentiation was proved by expression of osteocalcin, alkaline phosphatase activity (ALP) and mineralization was confirmed by using alizarin red (ARS). The quantity of cells was considerably increased in PCL/SF/HA/MH nanoparticles when compare to all other biocomposite nanoparticles and the cell interaction was observed more on PCL/SF/HA/MH nanoparticles. The electrosprayed PCL/SF/HA/MH biocomposite nanoparticle significantly initiated increased cell proliferation, osteogenic differentiation and mineralization, which provide huge potential for bone tissue engineering.

## 1. Introduction

The major obstacle faced by clinicians in orthopaedic and bone reconstructive surgery includes therapy for bone defects and fractures caused by tumour formation, trauma or diseases [1,2]. Autogenic and allogeneic bone implants are presently being used to repair fractures and improve bone growth in bone tissue engineering. However, grafting of massive implants has an inherent issue of slow curative level, cartilage development under poor vascularization and even causes apoptosis [3]. Biomaterials play an important role by providing appropriate substrates for cell growth and differentiation into the bone defect in addition to the structural and functional support for new tissue formation. The regeneration of bone deficiencies has reached some achievement when using injectable pastes and various scaffolding materials, there is a considerable space for development of new tissues in bone tissue engineering [4,5,6]. The potential benefits of nanoparticles are their average high surface to volume ratios, allowing better solubility thereby increasing bioactivity. The bioactive mesoporous nanoparticles will support cellular growth and bone repair, and also suitable for constructing macroporous devices to be useful in bone tissue regeneration [7].

Electrospray technique is one of the best methods to fabricate nano/microparticles. The basic principle in electrospraying technique is to set a high voltage to the biocomposite polymeric solution to flow out from the syringe in the form of particles. Advancement in electrospraying technology produced nanoparticles with more surface area-to-volume ratio and also physical and chemical properties more or less similar to the extracellular matrix (ECM) of natural bone. Human mesenchymal stem cells (MSCs) are multipotent cell source used for various biomedical applications, since they can be obtained from different sources such as bone marrow and adipose tissues [8,9]. MSCs have a multi-lineage differentiation potential leads to various cell types including osteoblasts, neuron-like cells, chondrocytes or fibroblasts [10,11].

Polycaprolactone (PCL) is a biodegradable polymer, generally used as an implantable material as it is easily degraded by hydrolysis of the ester linkages under biological environments [12]. Silk proteins are favourable materials for drug delivery and tissue engineering, due to their biocompatibility and biodegradability [13]. Silk fibroin (SF) protein is a Food and Drug Administration (FDA) approved naturally derived macromolecular protein that has been used to generate clinical sutures for several years [14]. Due to its tremendous tensile strength and biocompatibility, SF has been significantly used as a biomaterial in bone tissue engineering [15]. SF based tissue engineered bone transplants developed in bioreactors and fixed into calvarial deficiencies in mice revealed the ability to encourage bone formation within 30 days [16]. Consequently, the porous SF scaffolds supports the osteogenic-differentiation of MSCs, and such hMSCs cultured membranes have remarkably healed the femoral segmental defects in nude rats [17]. Hyaluronic acid (HA) is a naturally derived glycosaminoglycan used in biomedical applications such as post-surgical adhesion prevention and hydrophilic coatings. Addition of HA helps to increase cell proliferation and differentiation due to an increase in hydrophilicity and bone formation ability [18]. The combination of antibacterial agents with biomaterials is essential for repairing bone defects. Minocycline is a semi-synthetic, broad-spectrum bacteriostatic antibiotic, is energetic against aerobic, anaerobic, gram-positive and gram-negative bacteria, it possesses anti-collagenase activity, prevents bone infection and is able to promote proliferation and can improve bone growth, decrease connective tissue cessation and reduce bone resorption [19,20]. The present study, PCL was combined with SF, HA and Minocycline hydrochloride (MH), electrosprayed to obtain PCL/SF/HA/MH hybrid nanoparticles which has proved to be a promising biocomposite for MSCs proliferation, differentiation and mineralization holding great potential for bone tissue engineering.

## 2. Results and Discussion

### 2.1. Characterization of Nanoparticles

**Figure 1:**
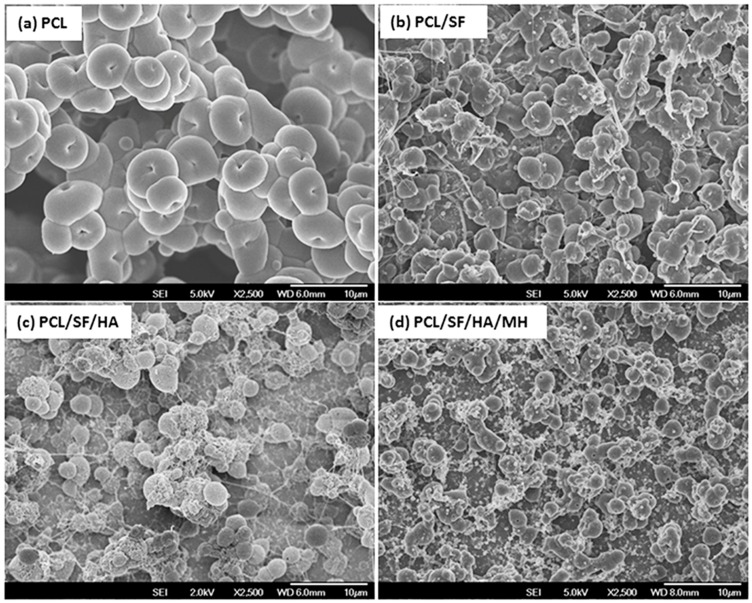
Field emission scanning electron microscope (FESEM) images of the electrosprayed micro/nanoparticles (**a**) polycaprolactone (PCL); (**b**) Polycaprolactone/silk fibroin (PCL/SF); (**c**) Polycaprolactone/silk fibroin/hyaluronic acid (PCL/SF/HA) and (**d**) Polycaprolactone/silk fibroin/hyaluronic acid/minocycline hydrochloride (PCL/SF/HA/MH) (0.54 ± 0.12 to 3.2 ± 0.18 µm). (Scale bar: 10 µm).

**Figure 2:**
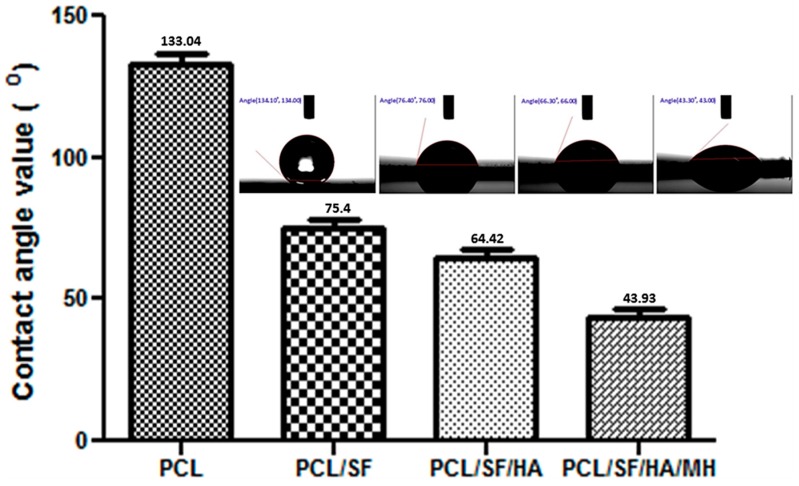
Water contact angle values of composite micro/nanoparticles.

The topography of nanoparticles plays an important role in the regulation of primary cell activities such as attachment and proliferation of cells in tissue engineering [21]. Field emission scanning electron microscope (FESEM) micrographs of nanoparticle showed nano-scaled and uniformed particles prepared under controlled conditions (Figure 1). The particle diameters were obtained in the range of 0.54 ± 0.12 to 3.2 ± 0.18 µm (Table 1). Contact angle measurement revealed that the addition of SF to PCL became hydrophilic because hydroxyl group in SF has the ability to form H bonds with H_2_O molecule for interpreting a significant increase in hydrophilicity as seen in PCL/SF with a contact angle of 75.4 ± 9.45° as compared to 133.1 ± 12.4° in PCL nanoparticles (Figure 2). Contact angle value of PCL/SF/HA/MH particles was significantly (*p* ≤ 0.001) reduced compared to pristine PCL nanoparticles. Upon addition of HA biomolecules, MH changes further hydrophilic with a contact angle of 64.42 ± 13.4 and 43.93 ± 30.8° for PCL/SF/HA and PCL/SF/HA/MH particles. The water absorbance ratio is directly proportional to the hydrophilic properties of the nanofibrous scaffolds which support prevention of dryness and the build-up of exudates on wounds [22].

**Figure 3:**
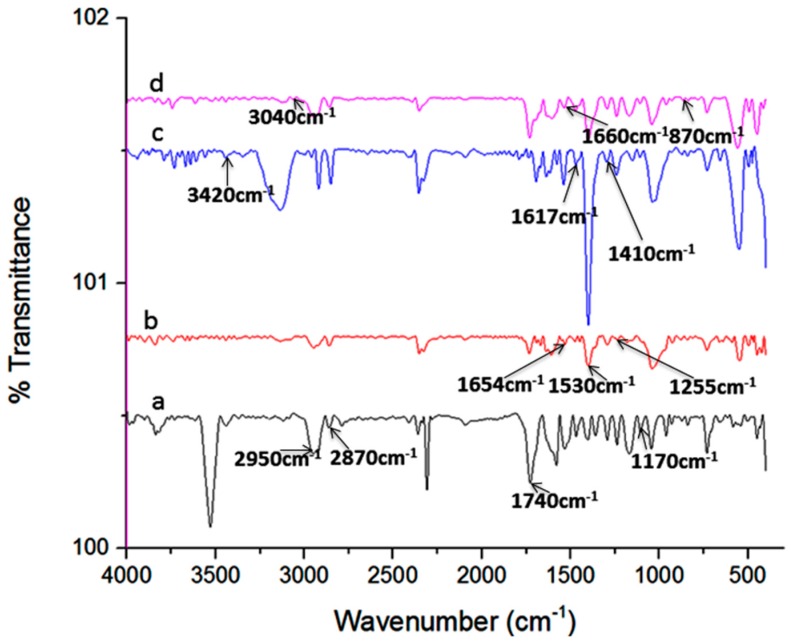
Fourier transforms infrared spectroscopy (FT-IR) study of (**a**) Polycaprolactone (PCL); (**b**) Polycaprolactone/silk fibroin (PCL/SF); (**c**) Polycaprolactone/silk fibroin/hyaluronic acid (PCL/SF/HA) and (**d**) Polycaprolactone/silk fibroin/hyaluronic acid/minocycline hydrochloride (PCL/SF/HA/MH) micro/nanoparticles.

The functional groups analyses of composite particles were studied using Fourier transform infrared spectroscopy (FT-IR) as presented in Figure 3. The peak characteristic of C–O–C symmetric stretching, C=O ester stretching, asymmetric and symmetric C–H alkane stretching on pristine PCL was observed at 1170, 1740, 2870 and 2950 cm^−1^ on the PCL. Furthermore, the characteristic peaks of amide I, II, and III of SF were also detected on the PCL/SF particles at 1654, 1530 and 1255 cm^−1^. The specific peak of hydrogen-bonded OH and NH stretching vibrations, asymmetric C=O and symmetric C–O stretching vibration of carboxyl groups in hyaluronic acid was noticed on PCL/SF/HA particle at 3420, 1617 and 1410 cm^−1^. The specific peak stretching vibration of amine, carboxyl group (C=O) and C–O stretching vibration of MH was detected at 3040, 1660 and 870 cm^−1^ on PCL/SF/HA/MH particles.

### 2.2. Particles Degradation and Minocycline Hydrochloride (MH) Release

**Figure 4:**
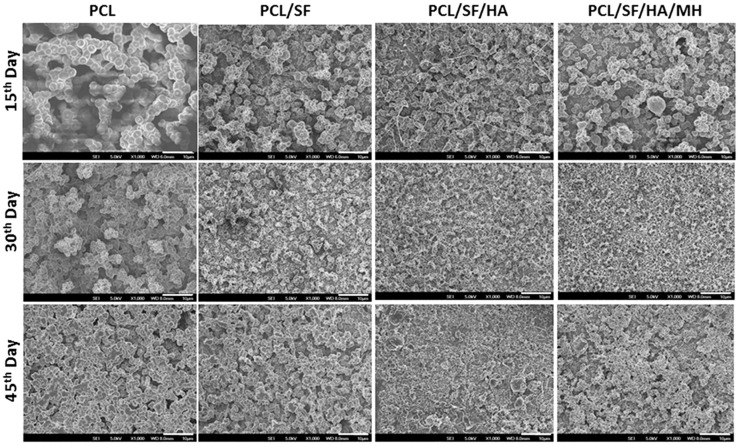
FESEM images show the degradation studies of biocomposite particles at various time points (Scale bar: 10 µm).

**Figure 5:**
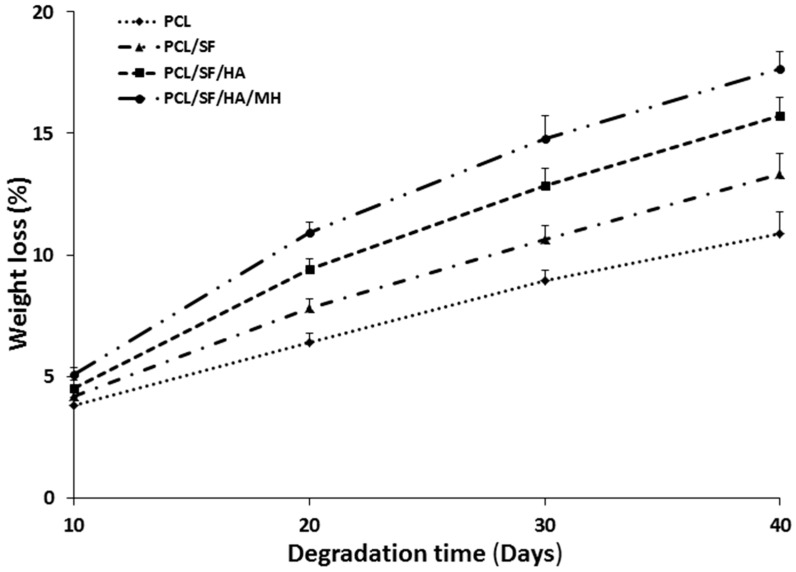
Biocomposite particles dry weight changes after degradation at various time points.

The degradation properties play an important role in biomolecule selection and strategy in tissue engineering [23,24,25,26]. Thus, the biocomposite scaffolds must meet definite model and suitable principles, with biocompatibility, tensile strength in particular cases of biomaterials. The ability of polymer biocomposites in a plethora of applications requires the optimal selection of matrix polymer chemistry, and matrix filler interaction for the degradation of materials [27]. The degradation of pristine PCL results from the hydroxylation and separation of excessive molecular mass chains, followed by altering carbon dioxide and H_2_O in the atmosphere of water. The structural variations of PCL, PCL/SF, PCL/SF/HA and PCL/SF/HA/MH particles on day 15, 30 and 45 are presented in Figure 4. After 15 days of degradation, for the PCL/SF, PCL/SF/HA, and PCL/SF/HA/MH particles and pore constructions remained similar and a small part of particles was scratch. The PCL/SF, PCL/SF/HA and PCL/SF/HA/MH nanoparticles were broken and finally, degenerate into small particles after day 30 and 45. This is caused by the hydrolysis and diffusion of small polymers from the samples, which effect in a disconnected construction and the diffusion of H_2_O molecule into the samples at initial degradation. Over a period of time the lengthy chains of polymers hydrolyzes and degrades the particles to breakdown into small parts. The weight loss was initiated by the degradation progress, which is evident that oligomeric substance able to dissolved in the medium from the polymer surface by the polymer chain hydrolysis. Degradation for 10–40 days of pristine PCL particles was 3.8% and 10.88% and the rate of degradation was much slower than PCL/SF particles (Figure 5). After day 30 and 40, addition of SF, the degradation rate PCL/SF, PCL/SF/HA and PCL/SF/HA/MH enriched and the weight loss was 10.65% and 13.33%, and 12.87% and 15.74%, and 14.8% and 17.65% respectively. Pristine PCL shows reduced rate of degradation then PCL/SF, PCL/SF/HA and PCL/SF/HA/MH particles. The crystallization of pristine PCL is greater as compared to electrosprayed PCL/SF, PCL/SF/HA and PCL/SF/HA/MH particles.

**Figure 6:**
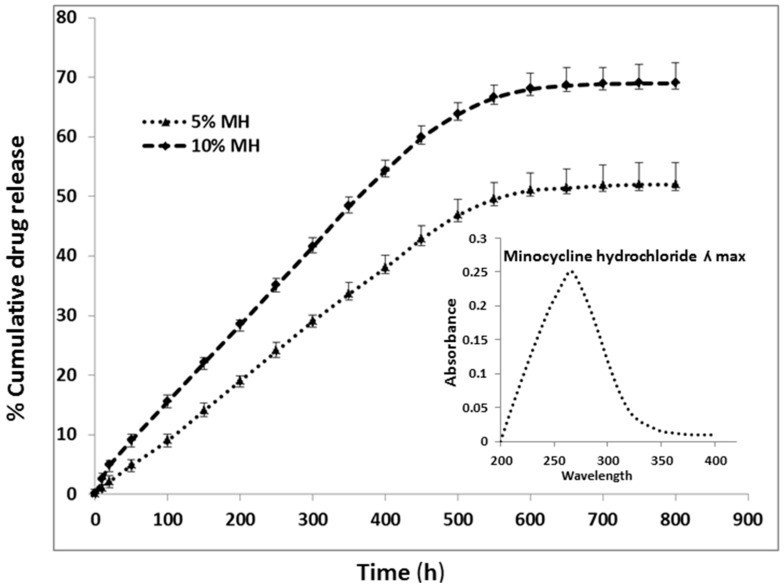
Drug release profile of minocycline hydrochloride in biocomposite nano/micro particles.

The even drug release profile of MH loaded biocomposite particles is shown in Figure 6. MH reveals λ_max_ at 260 nm, the drug release profile of MH were measured by drug-eluting membrane compositions of 5%, and 10% from the particles. Initial 100 h, burst releases were noticed about 10%–15%. A continuing sustained release was noticed within 150 and 500 h. The lower drug concentrations (0.04% and 0.07%), decreased release of drug was detected by the end of 30th day. The highest release ratio of MH was 5% and 10% *w*/*w* MH-loaded particles were around 52% ± 3% and 69% ± 5% at the end of 30th day. The progress of the experimental outcomes is the decrease of early burst release and the slow release due to the balance of drug inside the biocomposites structure. The initial burst released due to the influence of small amount of MH adsorbed on the sample surface.

### 2.3. Cell Morphology

**Figure 7:**
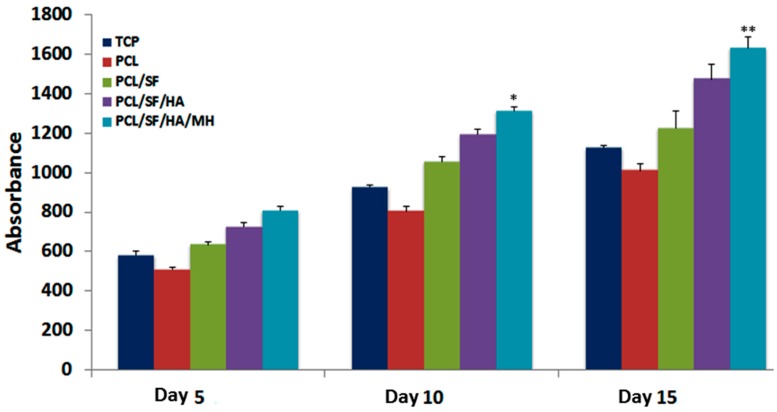
Proliferation analysis of mesenchymal stem cells (MSCs) on biocomposite particles on day 5, 10 and 15. * *p* ≤ 0.05, ** *p* ≤ 0.001.

**Figure 8:**
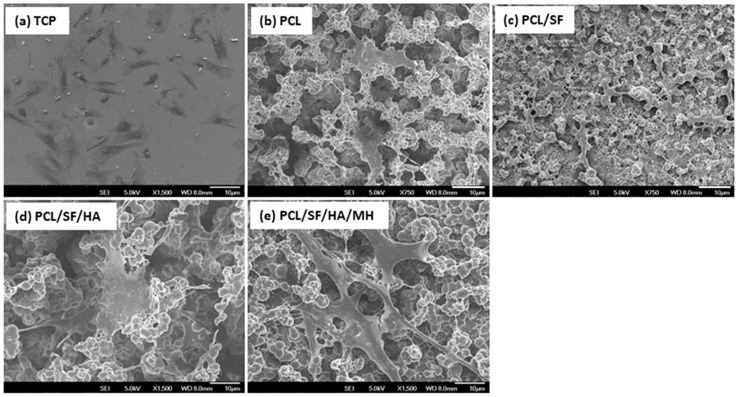
FESEM images showing the cell-biomaterial interactions on (**a**) TCP, (**b**) PCL (**c**) PCL/SF (**d**) PCL/SF/HA (**e**) PCL/SF/HA/MH micro/nanoparticles (Scale bar: 10 µm).

**Figure 9:**
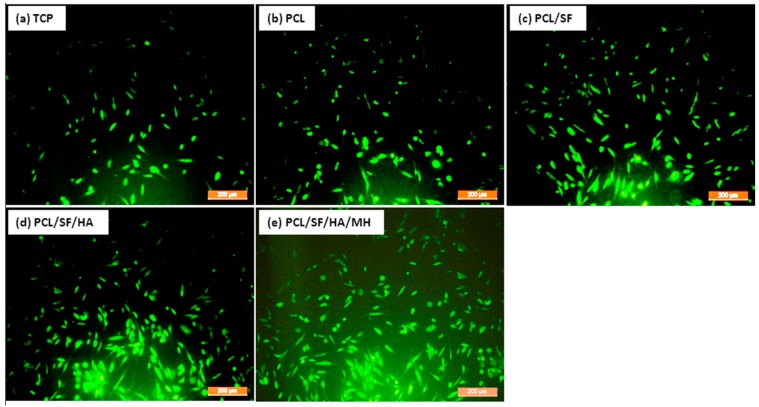
Cell morphology and 5-Chloromethylfluorescein diacetate CMFDA dye extrusion image to analyses the cell morphology on (**a**) TCP, (**b**) PCL (**c**) PCL/SF (**d**) PCL/SF/HA (**e**) PCL/SF/HA/MH micro/nanoparticles at 10× magnifications. The green color fluorescence indicate live cell morphology.

Scaffold properties are important determinants in regulating cell growth, morphology and cell signaling. Scaffold-cell interaction regulates early stage cellular activities namely cell adhesion and proliferation, which consequently influences differentiation and mineralization [28]. Cell culture after 5th, 10th and 15th days, the proliferation of MSCs was measured by (3-(4,5-dimethyl thiazol-2-yl)-5-(3-carboxymethoxyphenyl)-2-(4-sulfophenyl)-2*H*-tetrazolium inner salt) (MTS) assay (Figure 7). The rate of proliferation on PCL/SF/HA/MH samples was significantly increased (*p* ≤ 0.001) than PCL nanoparticles. The obtained results proved that the chemical alteration of the nanoparticle composition by adding SF/HA increased cell infiltration and proliferation. Hydroxyl groups and amino groups present in these samples served as ligands, which stimulated the differentiation of MSCs into osteoblasts. However, the rate of proliferation was higher on PCL/SF/HA/MH compared to all other particles after 15 days. This can be identified owing to the presence of HA, which gives better surface roughness and more surface range for greater cell attachment, proliferation and differentiation. FESEM image of MSCs (Figure 8) showed that the standard cell structure on all the samples with more mineral growth on the PCL/SF/HA/MH particles. Cellular function such as adhesion and proliferation showing the preliminary stages of cell-biomaterial interaction, finally leads to differentiation and mineralization [29]. Furthermore, the gradual adhesion of cells in to the nanoparticles was observed and experienced enhanced cell-to-cell interaction with the formation of filapodia structure. Cells cultured on to the nanoparticles revealed that filapodial interactions were capable of nano-topographical features and their cytoskeletal growth was essential for cell proliferation and differentiation into osteoblasts. The observed results proved that structure of the cells was comparatively uniform in all nanoparticles. However, cells on PCL/SF/HA/MH showed better osteoblast morphology and mineralization. This is because SF and MH initiate proliferation of cells and hyaluronic acid acts as a chelating agent, which results in the mineralization of osteoblasts required for bone tissue engineering. The live cell images in composite particles were determined by cell morphology and 5-Chloromethylfluorescein diacetate (CMFDA) dye expression method as shows in Figure 9. The normal cell morphology was observed in PCL/SF and PCL/SF/HA particles to prove the good environment for cell growth and mineralization. The MSCs, which underwent osteogenic differentiation, showed cuboidal morphology on PCL/SF/HA/MH nanoparticles unlike all the other samples.

### 2.4. Mineralization

**Figure 10:**
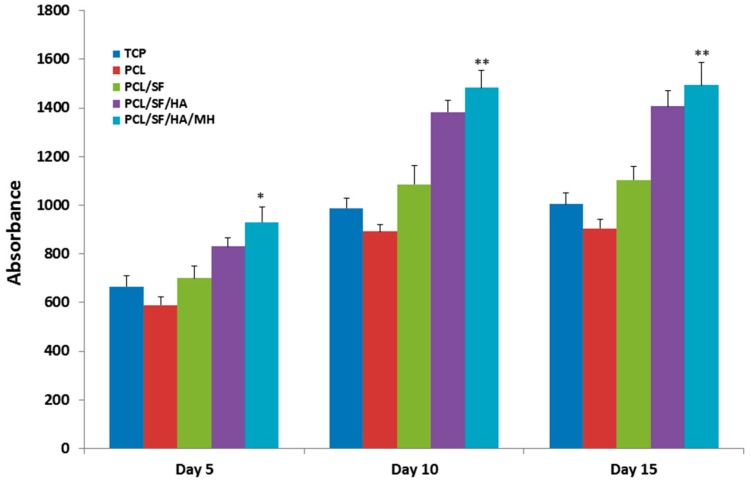
Alkaline phosphatase activity showing the osteogenic differentiation of MSCs on TCP, PCL, PCL/SF, PCL/SF/HA and PCL/SF/HA/MH micro/nanoparticles on day 5, 10 and 15. * *p* ≤ 0.05, ** *p* ≤ 0.001.

**Figure 11:**
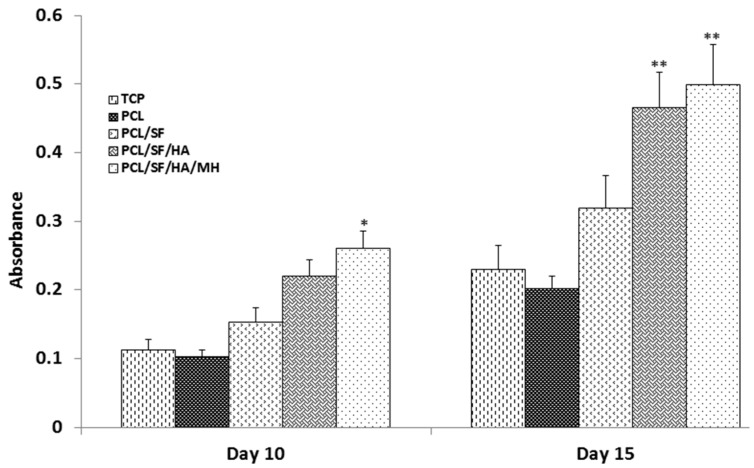
Quantification of mineral deposition in MSCs differentiated into osteogenesis. Alizarin Red-S staining on TCP, PCL, PCL/SF, PCL/SF/HA and PCL/SF/HA/MH micro/nanoparticles. * *p* ≤ 0.05, ** *p* ≤ 0.001.

**Figure 12:**
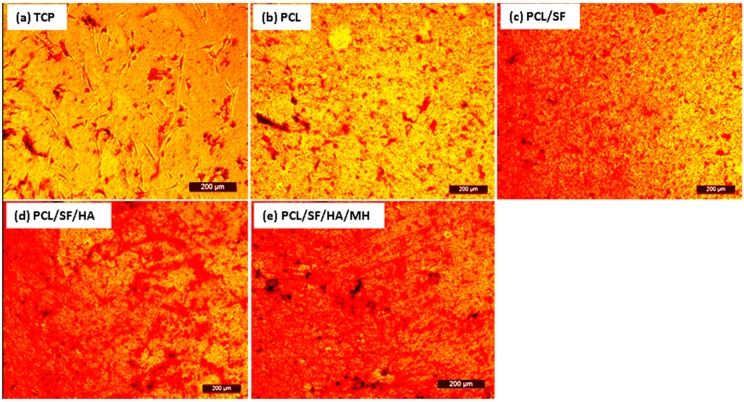
Optical microscopic images showing the secretion of minerals by osteogenic differentiation of MSCs. Alizarin red staining on day 15 in TCP (**a**), PCL (**b**), PCL/SF (**c**), PCL/SF/HA (**d**) and PCL/SF/HA/MH (**e**), (10× magnification).

An ideal composite particle should stimulate cell growth including both organic and inorganic constituents of natural bone materials. Alkaline phosphatase is an essential factor for bone matrix vesicles as it is involved in the production of apatite calcium phosphate and also a strong indicator of undeveloped osteoblast activity [30,31]. On day 15th, alkaline phosphatase activity (ALP) activity was significantly (*p* ≤ 0.001) increased in PCL/SF/HA/MH as compared to all other samples (Figure 10). This is because HA stimulates osteogenic differentiation of MSCs into new bone cells [32,33]. Alizarin red (ARS) staining showed a significant (*p* ≤ 0.001) increase of mineral deposition in PCL/SF/HA and PCL/SF/HA/MH compared to PCL on days 15 (Figure 11). Increased mineralization observed on day 10 and 15 of cell culture in PCL/SF/HA (22%, 46%), PCL/SF/HA/MH (26%, 49%), compared to PCL (10%, 20%). Qualitative analysis of mineralization was measured by alizarin red staining as shown in Figure 12. After 15th day of cells culture, PCL/SF/HA/MH (Figure 12e) interaction with cells showing more mineral deposition compared to PCL particles. Polylactic-co-glycolic acid attached HA/polyethylene glycol (PEG) scaffolds has effectively distributed Bone morphogenetic protein (BMP)-2 in vivo with gradual release from the scaffolds for up to a month. The developed tissue engineered porous scaffolds, which was later implanted in a confluent mouse osteoblastic cells (MC3T3-E1) sheet that provided desired cell growth for bone tissue engineering [34]. Presence of mineral deposits is a main indicator for matured osteoblasts that is useful for validating MSCs. Cell cultured on nanoparticles differentiated and reached mineralization level, where mineralized ECM is deposited on the particles. ALP activity indicates the presence of osteocalcin (OCN) expression during differentiation, because ALP positively regulates the production of ECM for mineral deposition [35,36]. Mineralization of MSCs is essential for the development of new bone and the observed results indicated that the addition of HA and MH enhanced mineralization, thereby improving mineral deposition for bone tissue engineering.

### 2.5. Expression of Osteocalcin

**Figure 13:**
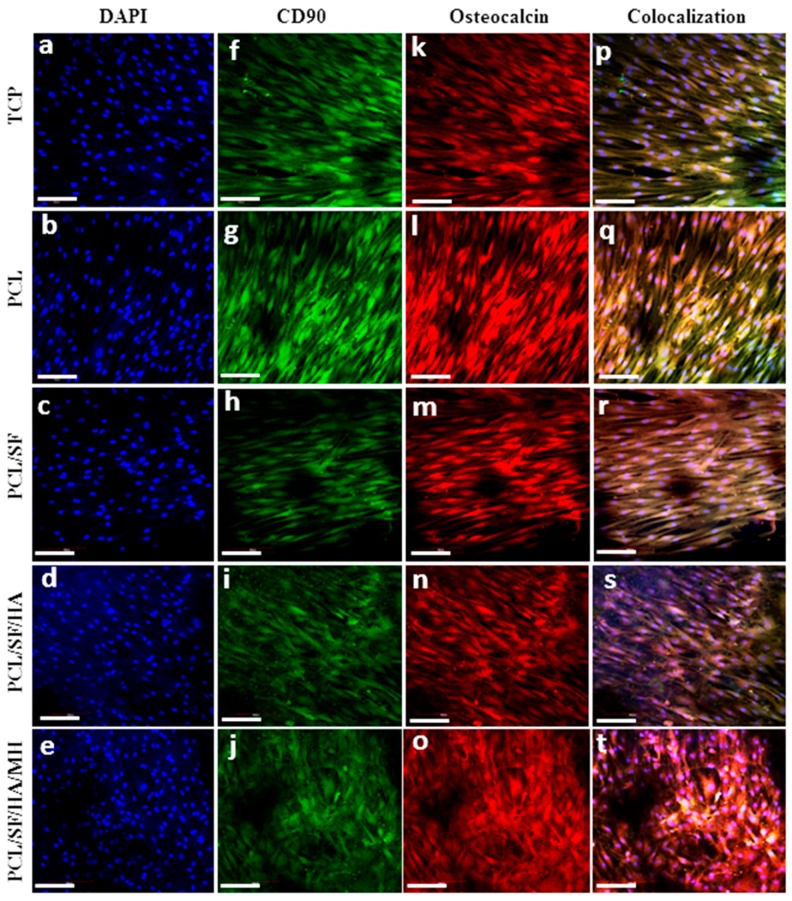
Confocal microscopy images to confirm the MSCs differentiation into osteogenesis. Nuclear staining DAPI (**a**–**e**) blue, MSCs specific marker protein CD90 (**f**–**j**) green, and osteoblasts specific marker protein osteocalcin (**k**–**o**) red. Merged images showing the dual expression of both CD90 and osteocalcin, characteristic of MSCs cells which have undergone osteogenic differentiation (**p**–**t**) on TCP (**a**,**f**,**k**,**p**), PCL (**b**,**g**,**l**,**q**), PCL/SF (**c**,**h**,**m**,**r**), PCL/SF/HA (**d**,**i**,**n**,**s**) and PCL/SF/HA/MH (**e**,**j**,**o**,**t**) with the nuclear staining by DAPI (**a**–**e**) at 20× magnification (Scale bar: 50 µm).

Osteocalcin (OCN) plays an important role in regulating the mineralization, because it contains glutamic acid-rich regions which bind strongly to Ca^2+^ [37]. Figure 13f–j showed that the expression of green colour which indicates cluster of differentiation (CD90) (MSC specific marker protein), then the cells differentiates into osteoblasts to express OCN protein Figure 13k–o. Cell nucleus were stained with 4′,6-diamidino-2-phenylindole (DAPI) indicate blue colour in Figure 13a–e. The obtained results showed that the osteogenic differentiation of MSCs by the dual expression of both CD90 and OCN in Figure 13d–t. The results showed that the MSCs seeded on PCL/SF/HA/MH exhibited the distinct cuboidal morphology seen in osteoblasts and increased levels of OCN expression indicate extensive differentiation into osteoblasts of more MSCs as compared to all other nanoparticles. The obtained results proved that the MSCs underwent osteogenic differentiation and mineralization, by the stimulation of HA in the biocomposite PCL/SF/HA/MH particles for bone tissue regeneration.

## 3. Materials and Methods

### 3.1. Materials

Polycaprolactone (PCL), hyaluronic acid, (HA) minocycline hydrochloride (MH), 1,1,1,3,3,3-hexafluoro-isopropanol (HFIP), methanol, Alizarin red-S, cetylpyridinium chloride were purchased from Sigma-Aldrich. Silk fibroin (SF) was purchased from Zhang Peng International Trading, Singapore. Dulbecco’s modified eagle’s medium (DMEM), nutrient mixture F-12, fetal bovine serum (FBS), antibiotics and trypsin-ethylene diamine tetra acetic acid (EDTA) were procured from GIBCO (Invitrogen, Carlsbad, CA, USA). CellTiter 96^®^ Aqueous one solution was obtained from Promega, Madison, WI, USA.

### 3.2. Fabrication of Nanoparticle

The electrospraying technique requires the optimization of several factors comprising high voltage power supply, distance between the needle tip and then collector plate, concentration of solutions and solution flow speed to make fine nanoparticles. Solutions of PCL, PCL/SF, PCL/SF/HA and PCL/SF/HA/MH were prepared for using electrospraying. 10% (*w*/*v*) solution of pristine PCL was prepared by using HFIP as a solvent. 10% (*w*/*v*) solution of PCL and SF (9:1) were prepared by using HFIP as a solvent. PCL/SF/HA and PCL/SF/HA/MH solutions were also prepared at the ratio of 8:1:1 and 7.5:1:1:0.5 in HFIP at the same concentration of 10%. For fabrication, solutions of PCL, PCL/SF, PCL/SF/HA and PCL/SF/HA/MH were separately fed into a 10 mL syringe connected to 22G × 1½ blunt needle and the needle was connected to a syringe pump at 1 mL/h flow rate with the high voltage of 16.0 to 16.5 kV. Under the influence of power supply the biocomposite solution was drained into particles, received on 15-mm cover slips, spread on a stainless steel plate enclosed with aluminium foil in 15 cm distance from the tip of the needle and later used for cell-culturing. The electrosprayed nanoparticles were dehydrated in vacuum pressure to remove any residual solvents.

### 3.3. Nanoparticles Characterization

Surface morphology of the nanoparticle was observed under field emission scanning electron microscope (FESEM, FEI-QUANTA 200F, Roche, Woerden, Netherlands) at an accelerating voltage of 10 kV, later the nanoparticles were sputter coated with platinum (JEOL JFC-1200 Auto fine coater, JEOL LTD, Tokyo, Japan). Each hybrid composite particles, 5 samples were randomly selected to measure the particle size using ImageJ software (National Institutes of Health, Dune Sciences, Inc. Eugene, OR, USA). The hydrophilicity of the electrosprayed nanoparticle was determined by sessile drop water contact angle analysis using video contact angle optima surface study system (AST Products, Billerica, MA, USA). Functional groups of electrosprayed nanoparticle were analyzed using Avatar 380 FTIR spectrometer (Thermo Nicolet, Waltham, MA, USA) to determine the presence of functional groups.

### 3.4. Degradation and Drug Release

Degradation studies of nanoparticles were conducted by in vitro method. The particles were weighed and kept in 15 mL of phosphate buffer solution (PBS) in an incubator at 37 °C. The PBS solution in the particle samples was changed once in two days. The degraded particles were washed carefully with H_2_O and then dehydrated through vacuum and their weights were measured. Weight loss ratio was measured by using the following formula.

$$\text{Weight loss }\%=\frac{W_{0}-{\text{W}}_{d}}{W_{0}}\;\times \;100\;\left(\%\right)$$

W_0_ is the actual weight before degradation; *W*_d_ is the dry weight after degradation. Structural modifications of particles before and after degradation were studied with FESEM at an accelerated voltage of 10 kV.

The minocycline hydrochloride (MH) release profile of MH-loaded PCL/SF/HA particle was analysed using PBS, the MH-loaded PCL/SF/HA particle (50 mg) were kept in a centrifuge tube then added 10 mL PBS as the release medium. For further study, the centrifuge tube was then kept in the incubator at 37 °C. At specific time points, 5 mL of solution was taken from the release medium and that volume was replaced with 5 mL PBS. Subsequently, the quantity of MH released at various time intervals up to 30 days was analysed using UV-visible spectrophotometer at 260 nm. Using the calibration curve of MH measured in the same condition, MH release percentage was determined and plotted as the curve versus time according to this equation:

$$\text{Release }\left(\%\right)=\frac{\text{Released MH}}{\text{Total loaded MH}}\;\times \;100\;\left(\%\right)$$

### 3.5. Cell Culture

Human mesenchymal stem cells (MSCs) (Lonza, Singapore) were cultured in DMEM/F12 (1:1) imbued with FBS in a 75 cm^2^ flask. MSCs were incubated at 37 °C in 5% CO_2_ for 7 days and every alternate day changed the medium. The confluences of cells were detached by adding trypsin with EDTA. Separated cells remained centrifuged and counted by Trypan blue assay using the hemocytometer. The coverslips were kept in well plates with stainless steel ring in each well to prevent swelling of the samples. The nanoparticles were then sterilized under UV light for 3–4 h, then dried with ethanol and then rinsed with PBS to eliminate remaining solvents. They were subsequently dipped in medium overnight incubated at 37 °C before cell culture. Cells were seeded on PCL, PCL/SF, PCL/SF/HA and PCL/SF/HA/MH at a concentration of 8 × 10^3^ cells per well.

### 3.6. Cell Proliferation

Cell proliferation was measured by using the colorimetric MTS assay (3-(4,5-dimethyl thiazol-2-yl)-5-(3-carboxymethoxyphenyl)-2-(4-sulfophenyl)-2*H*-tetrazolium inner salt) Cell Titer 96^®^ Aqueous One solution(Promega, Madison, WI, USA). The principle behind the estimation is that the mitochondria of metabolically energetic cells secretes dehydrogenase enzymes is responsible for the reduction of yellow tetrazolium salt in MTS to form purple colour formazan crystals. The colour exposed absorbance at 492 nm and the quantity of formazan formed is directly proportional to the amount of cells. Later the desired seeding periods of 5, 10 and 15 days, removed media from well plates and the samples were washed with PBS to remove any unattached cells followed by development with MTS mixture for 3 h at 37 °C in 5% CO_2_ incubator. Later, aliquots into 96 well plate and the solutions were analysed in a micro plate reader at 490nm (FLUOstar OPTIMA, BMG Lab Technologies Microplate Readers company, Ortenberg, Germany).

### 3.7. Cell Morphology

Cell morphology was examined after 15 days of MSCs culturing on to the samples using FESEM, removed the media and rinsed the substrate with PBS followed by 3% glutaraldehyde for 3 h to fix the cells. The samples were then washed with H_2_O and dried with ethanol in a series of concentration increases from 30%, 50%, 70%, 90% and 100% (*v*/*v*) sequentially for 15 min each. Lastly, the particles were dehydrated with hexamethyldisilazane and dried overnight. Then the samples were treated with platinum and cell morphology was analyzed using FESEM at an accelerating voltage of 10 kV. CMFDA fluorescent dye was used to observe the live cell morphology.

### 3.8. Alkaline Phosphatase Activity

Alkaline phosphatase activity (ALP) was measured by using alkaline phosphate yellow liquid substrate. Principle behind this reaction, ALP catalyzes the hydrolysis of colourless organic phosphate ester substrate, P-nitro-phenyl phosphate (pNPP) to yellow product p-nitrophenol and phosphate. After 5, 10 and 15 days of cell culture washed the samples with PBS followed by addition of 400 mL pNPP and then incubated for 30 min until the solution colour turns yellow. Stopped the reaction in addition of sodium hydroxide after which the solution was aliquot in 96-well plate and read in micro plate reader at 405 nm.

### 3.9. Mineralization

Alizarin red-S (ARS) dye binds specifically to calcium salts which are employed to measure and quantity of mineralization in differentiated osteoblasts. After 15 days of cell culture, washed the samples with PBS and fixed with chilled ethanol (70%) and washed with deionized water (DI) water and stained with ARS solution for 30 min. After three times rings with DI water, the samples were detected under the inverted optical microscope and images of the stain showing calcium deposition in Leica FW 4000 (version 1.0.2) (Leica Microsystems Imaging Solutions Ltd., Cambridge, UK). The stain was eluted with 10% cetyl pyridinium chloride for 60 min and the absorbance was measured at 540 nm in a spectrophotometer (Thermo Spectronic, Waltham, MA, USA).

### 3.10. Osteocalcin Expression

The differentiation of MSCs into osteoblasts was analyzed by using immunofluorescent staining of both MSC marker protein CD90 and osteoblast marker protein osteocalcin (OCN). After 15 days of culture, the samples were fixed with 4% paraformaldehyde and permeabilized with 0.5% Triton X-100. Non-specific binding spots were blocked with 3% BSA for 90 min. The biocomposite particles were treated with osteocalcin in the dilution of 1:100 for 90 min at 25 °C. In additional Alexa Fluor 594 secondary antibody was added in the dilution of 1:250 for 60 min. Then the samples were rinsed thrice with PBS to eliminate the additional staining and incubated using DAPI (1:3000) for 30 min. Then the samples were detached and fixed above the glass slide using Vectashield mounting medium (Vector lab, EON biotech PTE. Ltd., Singapore) and observed using fluorescent microscope (Olympus FV1000- Olympus Singapore PTE. Ltd., Singapore).

### 3.11. Statistical Analysis

Experiments were carried out in triplicates and the data shown were presented as mean ± standard deviation. Statistical differences were analyzed using student *t*-test and significance was considered at *p* ≤ 0.05.

## 4. Conclusions

The optimal combination of structural, biological and chemical properties of a bone implant plays a potential role in cell attachment and formation of new bone in bone tissue regeneration therapy. Nano-biomolecules can stimulate good cell adhesion, production of ECM and mineralization. PCL/SF/HA/MH nanoparticles fabricated by electrospraying method and MSCs grown on them showed greater cell proliferation and increased ALP activity as compared to all other particles. Osteogenesis was also induced on these biocomposite particles with maximum osteocalcin expression and subsequent mineralization. The electrosprayed PCL/SF/HA/MH hybrid nanoparticles harness abundant potential for cell attachment, proliferation, differentiation and subsequent mineralization of MSCs for bone tissue engineering.

## Figures and Tables

**Table 1:** Particle size measurement of biocomposite nanoparticles.

Composite Particles	Particle Size (µm)
Polycaprolactone (PCL)	3.2 ± 0.18
Polycaprolactone/silk fibroin (PCL/SF)	1.62 ± 0.59
Polycaprolactone/silk fibroin/hyaluronic acid (PLC/SF/HA)	0.9 ± 0.15
Polycaprolactone/silk fibroin/hyaluronic acid/minocycline hydrochloride (PCL/SF/HA/MH)	0.54 ± 0.12
